# Necessity of Fine-Needle Aspiration in Probably Benign Sonographic Appearance of Thyroid Nodules

**DOI:** 10.22038/ijorl.2021.53741.2831

**Published:** 2021-07

**Authors:** Aida Sharifi Haddad, Behzad Aminzadeh, Seyed Ali Alamdaran, Shokoufeh Bonakdaran, Amirhosein Jafarian, Samira Rahemi, Negar Morovatdar, Ramesh Giti

**Affiliations:** 1 *Department of Radiology, Faculty of Medicine, Mashhad University of Medical Sciences, Mashhad, Iran. *; 2 *Surgical Oncology Research Center, Mashhad University of Medical Sciences, Mashhad, Iran. *; 3 *Department of Internal, Faculty of Medicine Mashhad University of Medical Sciences, Mashhad, Iran. *; 4 *Department of Pathology, Faculty of Medicine Mashhad University of Medical Sciences, Mashhad, Iran.*; 5 *Clinical Research Units, Faculty of Medicine, Mashhad University of Medical Sciences, Mashhad, Iran.*

**Keywords:** FNA, Malignancy, Thyroid nodule, TI-RADS

## Abstract

**Introduction:**

The management of thyroid nodules has been proposed based on US features and information obtained from Fine-Needle Aspiration Cytology (FNAC). In this study, we checked the diagnostic value of ultrasound in comparison with FNAC in probably benign nodules of thyroid.

**Materials and Methods:**

Patients with thyroid nodules referred to the Radiology department from 2015 to 2020, were classified into five types based on the American College of Radiology/thyroid imaging reporting and data system (TI-RADS) standards. The patients with TI-RADS III-V were examined by FNA biopsy. Subsequently, the collected data of 535 patients having thyroid nodules with TI-RADS III were statistically analyzed.

**Results:**

The mean age of the patients was estimated at 46.57. The analysis of TI-RADS III cases examined by the FNA biopsy revealed that 99.1% of the cases were diagnosed with benign lesions. The mean size of benign and malignant nodules was 27mm and 41mm, respectively. There was no significant correlation between the size of the nodules or patients age, and thyroid malignancy (P-values > 0.05).

**Conclusion:**

There was a very low chance of malignancy (0.9%) in thyroid nodules with thyroid imaging classification of TI-RADS III. Furthermore, no meaningful correlation was observed between the size of the nodules and their malignancy. Therefore, the use of FNAC, based on the current guidelines, on thyroids for nodules larger than 2.5 cm might need to be revised.

## Introduction

Thyroid nodules are highly prevalent in different societies with a prevalence rate of 5% and 1% among women and men, respectively, in societies with adequate iodine intake (1,2). Fine-Needle Aspiration Cytology (FNAC) is one of the most accurate methods for the diagnosis of malignant thyroid nodules (3,4). Although FNAC is an effective method to distinguish malignant from benign nodules, it is impossible to diagnose all referred patients using this method since it is an invasive, relatively costly, time-consuming, and challenging approach, especially in benign lesions (5-7). 

Additional diagnostic procedures, including nuclear scan, has been recommended in some guidelines to these patients (5). Ultrasound results have been confirmed by various researchers for differentiation of benign from malignant thyroid tumors. Papers have been recently presented pointing to the application of these findings to classify and differentiate benign and malignant lesions, which often express the probability of malignancy with high precision (1,6-10). 

Nowadays, the most popular medical guidelines state the diagnostic approach based on ultrasound appearance and mass size. A majority of these guidelines recommend FNAC in all solid masses larger than 2.5 cm, even for those with a completely benign appearance. 

Ultrasound follow-up has been suggested for smaller benign nodules within 2 years and then at intervals of 3-5 years (11). 

Considering the high prevalence of benign thyroid nodules in nodules larger than 2.5 cm, and concerning their almost specific ultrasound features, especially based on thyroid imaging reporting and data system (TI-RADS) or U-classification, this study was performed to evaluate the prevalence of malignancy in nodules of the thyroid gland with probably benign ultrasound features (i.e., TI-RADS III) having FNAC indication due to the large size of nodules. 

Therefore, in this study, we checked the diagnostic value of ultrasound in comparison with FNAC in these probably benign lesions and, define the relationship between the size of these nodules with the prevalence of malignancy.

## Materials and Methods

To conduct this retrospective research, the researchers collected sonography, cytology, pathology, and follow-up data of patients with thyroid nodules who referred to the ultrasonography wards related to Mashhad medical university of science during 2015-2020. There were no age and gender limits as an input criterion.

The ultrasonography of thyroid nodules was examined by a high-resolution gray-scale Esoate Class C or Samsung H60 device with 8-16 MHz probes in each patient. The study variables included the main characteristics of a thyroid nodule, such as size, shape, margin, structure, echogenicity, heterogeneity, echogenic dot, expansion to the capsule, vertical axis position, and nodular vascularity, which were obtained by sonographic evaluation. According to these variables and American College of Radiology (ACR)/ TI-RADS criteria, thyroid nodules were classified as follows: TI-RADS I: normal thyroid gland; TI-RADS II: benign thyroid lesions (cysts, adenomatous goiter, and thyroiditis); TI-RADS III: probably benign thyroid lesions; TI-RADS IV: suspected malignant thyroid disease; and TI-RADS V: malignant thyroid lesions. Following the allocation of patients according to TI-RADS classification, TI-RADS II patients underwent an average follow-up period of 2 years, and patients suspected of malignancy (groups III to V) were subjected to FNA based on the guideline.

A 25-gauge needle was used for aspiration from nodules, which were smeared directly onto 4-6 glass slides and stained with May-Grunwald-Giemsa. All specimens were examined by an expert cytopathologist. Cytopathologic report was based on Bethesda classification system. For nodules without FNAC indication according to guidelines, the patients were followed up for 2 years and were considered to have benign if the nodule size and/or TI-RADS grade did not increase. 

**Fig 1:**
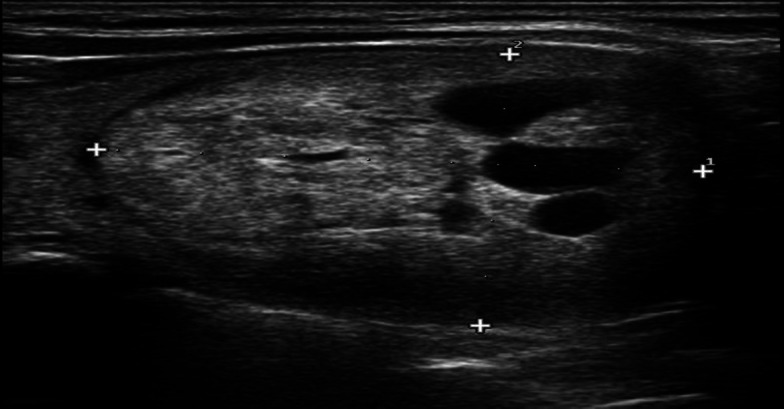
Ultrasonographic appearance of TI-RADS III nodule; they are solid or almost solid, iso-, or hyperechoic, well-defined oval shape nodule without echogenic foci

Afterward, cytopathology or follow-up results of thyroid nodules with a probably benign view (i.e. TI-RADS III), were selected and statistically analyzed according to ultrasound features. Category TI-RADS III includes almost solid, iso-, or hyperechoic, well-defined oval shape nodule without echogenic foci (Fig 1). 

Patients in other TI-RADS categories and those whose information was not properly retrieved at each stage of sample collection were excluded from the study. Statistical analyses were performed in SPSS software (version 16; SPSS Inc. Released 2009. PASW Statistics for Windows, Chicago: SPSS Inc.). Continuous variables were expressed as mean ± SD and categorical variables as numbers and percentages. The Chi-Square test was used to examine the relationship between qualitative variables and p-values of <0.05 were considered as the significant level in all tests.

## Results

**Fig 2:**
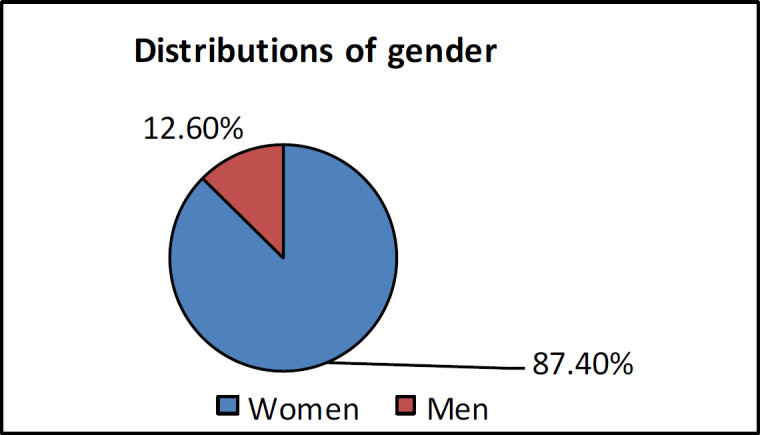
Distribution of gender

In this study, among the 535 patients in the TI-RADS III class, 87.4% and 12.6% of the cases were women and men, respectively (Figure 2). The mean age of the studied population was 46.57 years (range: 1-90 years). There was no significant relationship between the age of patients and the benign or malignant nature of the nodule (P=0.109). In most patients (48.3%), a single nodule was seen in the thyroid gland, while in 38.6% of patients, more than three nodules were observed in the thyroid. The prevalence of malignancy decreased with an increase in the number of thyroid nodules (P=0.001).

The association between nodule size and benign or malignant nature of nodule was evaluated, which showed no significant relationship (P=0.323). The mean scores of nodule size in benign and malignant cases were estimated at 26.93±17.067 and 41.00±29.462 mm in the range of 10-97 and 23-75 mm, respectively.

**Fig 3:**
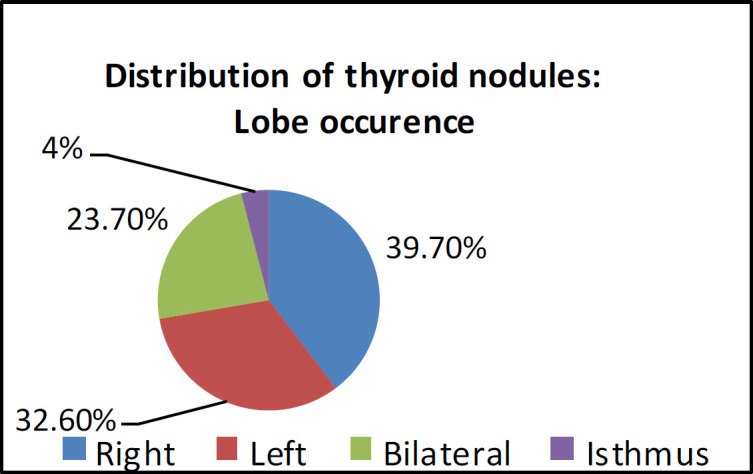
Distribution of thyroid nodules: lobe occurrence

The distribution of thyroid nodules was as follows: right lobe (39.7%), left lobe (32.6%), bilateral (23.7%), and isthmus (4%) (Figure 3). In total, 67.9% and 32.1% of nodules were unilateral and bilateral, respectively. The prevalence of malignancy was lower in cases that had nodules in both lobes (P=0.15).

With respect to the appearance of thyroid lobes, 84.6% of the patients had a normal appearance, while 14.1%, 6%, 4%, 3%, and 1% of the subjects had thyroiditis, multinodular goiter, combined thyroiditis and atrophic thyroid appearance, atrophic appearance, and combined thyroiditis and goiter appearance, respectively. There was no significant correlation between malignancy and sonographic features of the thyroid gland. In the study of follow-up and needle aspiration of class 3 thyroid nodules, 99.1% of the cases had benign lesions, including adenomatoid goiter, colloidal goiter, multinodular goiter, thyroiditis, and colloidal cysts, and less than 1% (n=5) of the patients had malignant lesion (papillary thyroid carcinoma).

## Discussion

The size of thyroid nodule is a feature of ultrasound that has been evaluated in various guidelines and studies, including ours. Although the size of thyroid nodule is not an indicator of FNA by itself, it has an important role in determining the necessity of FNA in different guidelines (12).

Nowadays, in ACR guidelines, there is no recommendation of FNA for any TI-RADS I and II nodule independent of nodule size. No significant correlation was found between the increased risk of malignancy and tumor size in benign-appearing nodules in ultrasound, which was consistent with the ACR guideline in TI-RADS II nodules (10). 

The results of our study indicated that there was no association between the size of nodule and risk of malignancy in TI-RADS III. Currently, ACR recommends that FNA should be performed on nodules with TI-RADS III larger than 2.5 cm (10). However, in the present study, no relationship was observed between the size of nodule and risk of malignancy in this group of nodules. Moreover, the risk of malignancy in nodules with TI-RADS III was revealed to be very low (less than 1%). 

Nevertheless, these results were inconsistent with those of studies reporting a relationship between the increasing size of the nodule and the raising risk of malignancy in thyroid nodules regardless of nodule appearance in ultrasound. A significant relationship was found between thyroid nodule size and malignancy in some studies (13-15). Based on the findings of research conducted in 2013, there was a significant relationship between an increase in nodule size and an increase in the risk of malignancy in nodules less than 2 cm (14). The results of a meta-analysis performed in 2016 showed that nodules with a size greater than 3 cm and less than 6 cm had the greatest association with malignancy (15). In a systematic review performed in 2015, a significant correlation was found between nodules larger than 30 mm and the risk of malignancy (13).

Although the results of these studies are inconsistent with those of our research, it should be noted that in a majority of the mentioned investigations, all thyroid nodules were studied regardless of benign or malignant features of nodules in sonography, whereas the current research merely studied nodules with probably benign sonographic views. Therefore, there is the likelihood of no correlation between the size and probability of malignancy in nodules with a probably benign appearance in ultrasound, and the association mentioned in previous studies may have been only due to such a relationship in nodules with a malignant appearance.

It should be noted that the results of some other studies indicated that there was no significant relationship between nodule size and its malignancy (16, 17), which were consistent with the results of the present study. In a study carried out in 2008, no correlation was found between the size of the nodule and malignancy, and the authors recommended that the nodule size should not be an indication to perform FNA (16). The researchers of another study (2012) found no association between nodules larger than 4 cm and malignancy risk (17). Furthermore, in a study in 2017, a significant inverse relationship was found between nodule size and malignancy, and it was noted that the highest probability of malignancy was related to nodules with a size less than 20 mm, and in nodules larger than 20 mm, the risk of malignancy did not change significantly with changing the size of nodules (18). In a study performed by Hong in 2018, the size of the nodule was measured in three groups with high, low, and moderate risk, the results of which showed that there was no relationship between nodule size and malignancy in nodules with a high risk of malignancy. However, the risk of malignancy was slightly increased with an increase in the size of low or moderate risk nodules. In the mentioned study, in contrast to the 25-mm-cut-off of ACR, a 30 mm cut-off was considered to present a large nodule (19). Regarding the aforementioned studies, there is still controversy about the relationship between nodule size and the possibility of malignancy.

It is highlight that the cytological confirmation of lesions in various nodules could not be achieved, especially in those with a size of less than 1 cm (20). However, In all thyroid nodules with different TI-RADS groups, there may be a significant correlation between size and malignancy.

Nonetheless, according to the findings of this study, 99.1% prevalence of benign nodules was reported in thyroid nodules with TI-RADS III, while only 0.9% of nodules were malignant, indicating no significant association between nodule size and benign or malignant nature of tumor in this category of nodules. This means that if the procedure is conducted according to the guideline, it is required to perform FNA for most patients with benign-appearing nodules in sonography, while 99.1% of their results are consistent with the initial diagnosis of ultrasound and FNA only imposes high costs to patients and healthcare system. One of the limitations of the present study was related to the low number of malignant cases. However, the cause of this low frequency was attributed to the low prevalence of malignancy in TI-RADS III nodules in our population. In this study, the relationship between changes in size and malignancy risk in nodules with a higher risk of malignancy was not evaluated. Therefore, it is recommended to perform such a study to facilitate the analysis of the association between nodule size and malignancy risk in TI-RADS III nodules. Another limitation of the current research was the lack of any information on the prevalence of benign-appearing nodules in our local population. Regarding, the prevalence evaluation of benign nodules in our population could help determine the reason for the absence of a significant relationship in our research. For example, in populations with a high prevalence of benign nodules, it is expected to observe more nodules with TI-RADS III showing a benign FNA result. In addition, studies by grouping these nodules based on the presence of symptoms of adenomatoid goiter, thyroiditis (white knight nodule), etc. may lead to more accurate results. Considering the results of this study and the low prevalence of malignancy in thyroid nodules with benign or possibly benign sonography appearance, which are classified in stage III based on TI-RADS, it can be concluded that a stage III thyroid nodule can be followed up by ultrasound without a need for FNAC. Due to the lack of significant correlation between malignancy and nodule size, the use of ultrasound follow-up can be purposed even for nodules larger than 2.5 cm; however for now, FNA is advised for nodules larger than 2.5 cm in thyroid guidelines regardless of the ultrasound view.

## Conclusions

In conclusion, according to the results of this study and given the high prevalence (99.1%) of a benign outcome in probably benign thyroid nodules in ultrasound that are classified in TI-RADS stage III, very low prevalence of malignancy (0.9%) in these nodules, and the lack of significant correlation between the prevalence of malignancy and nodule size or patients age in this group, it appears that FNAC recommendation according to thyroid guidelines in well defined iso- or hyperechoic oval nodules larger than 2.5 cm is questionable and demands more studies.
